# Metastatic medullary thyroid carcinoma: a new way forward

**DOI:** 10.1530/ERC-21-0368

**Published:** 2022-04-26

**Authors:** Anna Angelousi, Aimee R Hayes, Eleftherios Chatzellis, Gregory A Kaltsas, Ashley B Grossman

**Affiliations:** 1 Unit of Endocrinology, First Department of Internal Medicine, Laiko Hospital, National and Kapodistrian University of Athens, Athens, Greece; 2 Neuroendocrine Tumour Unit, ENETS Centre of Excellence, Royal Free Hospital, London, UK; 3 Endocrinology Diabetes and Metabolism Department, 251 Hellenic Air Force and VA General Hospital, Athens, Greece; 4 First Department of Propaedeutic Internal Medicine, Laiko Hospital, National & Kapodistrian University of Athens, Athens, Greece; 5 Green Templeton College, University of Oxford, Oxford, UK; 6 Centre for Endocrinology, Barts and the London School of Medicine, London, UK

**Keywords:** medullary thyroid cancer, treatment, tyrosine kinase inhibitors, selpercatinib, pralsetinib, PRRT, immunotherapy

## Abstract

Medullary thyroid carcinoma (MTC) is a rare malignancy comprising 1–2% of all thyroid cancers in the United States. Approximately 20% of cases are familial, secondary to a germline *RET* mutation, while the remaining 80% are sporadic and also harbour a somatic *RET* mutation in more than half of all cases. Up to 15–20% of patients will present with distant metastatic disease, and retrospective series report a 10-year survival of 10–40% from time of first metastasis. Historically, systemic therapies for metastatic MTC have been limited, and cytotoxic chemotherapy has demonstrated poor objective response rates. However, in the last decade, targeted therapies, particularly multitargeted tyrosine kinase inhibitors (TKIs), have demonstrated prolonged progression-free survival in advanced and progressive MTC. Both cabozantinib and vandetanib have been approved as first-line treatment options in many countries; nevertheless, their use is limited by high toxicity rates and dose reductions are often necessary. New generation TKIs, such as selpercatinib or pralsetinib, that exhibit selective activity against *RET*, have recently been approved as a second-line treatment option, and they exhibit a more favourable side-effect profile. Peptide receptor radionuclide therapy or immune checkpoint inhibitors may also constitute potential therapeutic options in specific clinical settings. In this review, we aim to present all current therapeutic options available for patients with progressive MTC, as well as new or as yet experimental treatments.

## Introduction

Medullary thyroid carcinoma (MTC) is a malignant neuroendocrine tumour originating from the parafollicular or C-cells of the thyroid, capable of secreting calcitonin and carcinoembryonic antigen (CEA) (Ceolin *et al.* 2019). It is a rare malignancy comprising 1–2% of all thyroid cancers in the United States (Tuttle *et al.* 2014, Lim *et al.* 2017). Approximately 20% of cases are familial, secondary to a germline rearranged during transfection (*RET*) mutation, while the remaining 80% are sporadic (Bai *et al.* 2020) and also harbour a somatic *RET* mutation in more than half of all cases, with *RET* M918T being the most frequent genetic alteration encountered in 30–50% of aggressive sporadic MTC (Dvorakova *et al.* 2008, Taccaliti *et al.* 2011).

### Initial therapeutic approach to MTC

The standard treatment for MTC is total thyroidectomy and dissection of cervical lymph node compartments, as surgery is the only curative treatment (Wells *et al.* 2015). Patients with persistent or recurrent MTC localised to the neck are candidates for repeated neck explorations. However, in the presence of widespread regional or metastatic disease, extensive surgery is not associated with a higher cure rate or survival benefit and should be considered mainly for local symptom control (Wells *et al.* 2015, Randle *et al.* 2017).

Post-operative biochemical remission of serum calcitonin is significantly correlated with an improved 5-year recurrence rate but not 5-year survival (Jung *et al.* 2016). The 5-year and 10-year survival rates are 25 and 8%, respectively, when the doubling time of calcitonin is less than 6 months, and 92 and 37%, respectively, and when the doubling time ranges from 6 months to 2 years (Wells *et al.* 2015). Currently, a short calcitonin and CEA doubling time (<6 months) are considered the best available indicators to assess tumour behaviour, MTC recurrence and cancer mortality (Barbet *et al.* 2005, Laure Giraudet *et al.* 2008). Post-operative levels of serum calcitonin > 150 ng/L generally indicate distant metastases (Laure Giraudet *et al.* 2008).

Neck and/or chest computerised tomography (CT), hepatic magnetic resonance imaging (MRI) and bone scintigraphy are the main imaging modalities utilised for MTC staging and follow-up (Maia *et al.* 2014, Wells *et al.* 2015). Although several radionuclide imaging modalities are available, positron emission tomography (PET) CT using ^18^F-fluorodeoxyglucose (^18^F-FDG), ^18^F-DOPA and ^68^Ga-DOTA-somatostatin analogues (^68^Ga-SSA) offer higher sensitivity compared with conventional imaging (CT/MRI). Of interest, a study evaluating the performance of ^68^Ga-1,4,7,10-tetraazacyclododecane-1,4,7,10-tetraacetic acid-octreotate (DOTATATE)-PET/CT in detecting MTC lesions suggested that it is highly sensitive in identifying bone metastases and could be a substitute for bone scans, and importantly, as a theranostic, it indicates a potential therapeutic tool in the presence of considerable uptake (Castroneves *et al.* 2018, Hayes *et al.* 2021).

### Metastatic disease

Up to 15–20% of patients will present with distant metastatic disease at diagnosis, and retrospective series report a 10-year survival of 10–40% from time of first metastasis (Roman *et al.* 2006, Chiacchiarini *et al.* 2021).

Cytotoxic chemotherapy, as well as local treatments such as external beam radiotherapy, has only a limited and short-term benefit (Wells *et al.* 2015). Currently available systemic treatments include the use of multi-tyrosine kinase inhibitors (TKIs), which partially inhibit multiple kinases including RET, and often impair multiple signalling pathways (Filetti *et al.* 2019). TKIs have shown prolonged progression-free survival in patients with tumour progression, although at a cost of considerable toxicity (Elisei *et al.* 2013). Although the Food and Drug Administration (FDA) and European Medicines Agency (EMA) have approved several TKIs for treating advanced MTC, novel therapeutic modalities are still required to treat patients who fail to respond to TKIs or experience unacceptable toxicity. New-generation targeted therapies such as immunotherapy and peptide receptor radionuclide therapy (PRRT) may be promising but require prospective randomised trials.

In this review, we aim to present all current therapeutic options available for patients with progressive MTC, as well as the new or as yet experimental treatments based on the individualised molecular profile of each patient.

## Therapeutic strategies for metastatic MTC

When evaluating a patient with advanced MTC, various factors need to be taken into account. Is the patient symptomatic or asymptomatic? Is the locoregional disease controlled? Is there any calcitonin-related secretory syndrome or ectopic adrenocorticotropic hormone (ACTH) syndrome? Are there lesions that require intervention due to an imminent risk or associated symptoms? What is the rate of progression (calcitonin doubling-time and imaging findings based on Response Evaluation Criteria in Solid Tumors (RECIST) criteria)?

### Tyrosine kinase inhibitors

**Figure 1:**
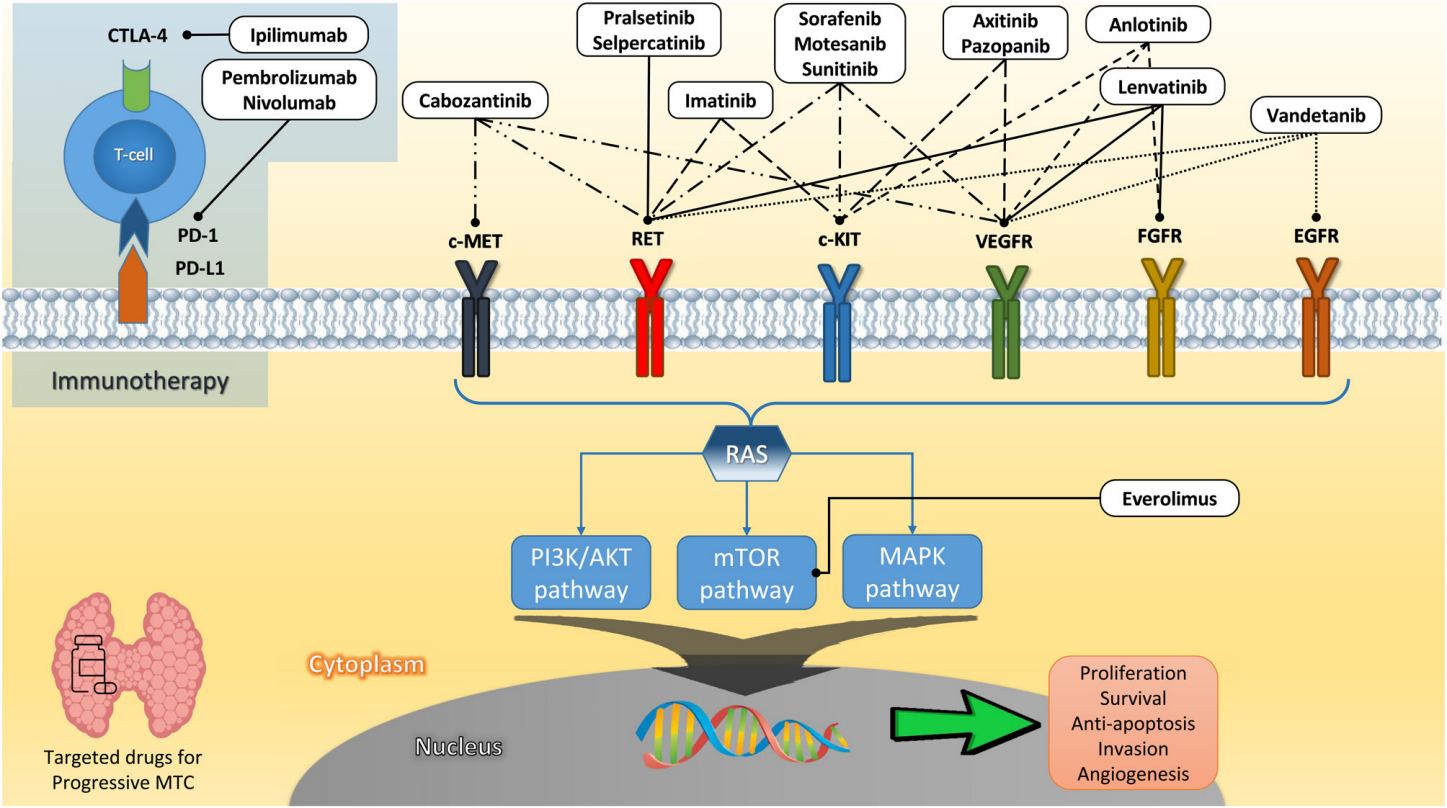
Regulating pathways of MTC and MTC-targeted therapy (potential and confirmed agents) with their corresponding targets and receptors.

Advances in understanding the molecular mechanisms and intracellular signalling pathways involved in MTC pathogenesis have allowed for the development of targeted therapies, offering new perspectives on effective therapies for advanced MTC. Generally, these therapies are based on the blockade of the MAPK and RAS pathways, which promote MTC development. Many compounds have been developed to inhibit the activity of these pathways (Fig. 1).

#### Multikinase inhibitors

Tyrosine kinase inhibitors (TKIs) provide a therapeutic benefit in cancer by blocking tyrosine kinase-dependent oncogenic pathways. Several multitargeted TKIs have been tested in MTC, including motesanib (Schlumberger *et al.* 2009), sorafenib (de Castroneves *et al.* 2016), sunitinib (Carr *et al.* 2010), axitinib (Cohen *et al.* 2008, 2014), imatinib (de Groot *et al.* 2007), pazopanib (Bible *et al.* 2014), anlotinib (Sun *et al.* 2018), lenvatinib (Haugen *et al.* 2016), vandetanib (Wells *et al.* 2012) and cabozantinib (Elisei *et al.* 2013) (Table 1). Of these various TKIs, only vandetanib and cabozantinib are currently FDA and EMA approved for treating unresectable and progressive advanced MTC (Wells *et al.* 2015, Schlumberger *et al.* 2017). Because patients with progressive MTC may follow a relatively indolent disease course and can experience prolonged survival even without therapy, prospective trials should preferably mandate RECIST progression on trial enrolment and use progression-free survival (PFS) along with overall survival (OS) to evaluate drug efficacy (Hadoux & Schlumberger 2017).

**Table 1:** Clinical trials of multikinase inhibitors in medullary thyroid cancer.

TKIs	Phase	Population (*n*)	Studied groups	Target of action	Median PFS, (HR (95% CI), months),*P*	Median OS (HR (95% CI), months), *P*	AEs	FDA approval
Vandetanib (ZETA study) (NCT00410761)	3	Advanced or metastatic MTC (*n* = 331)	Vandetanib-treated MTC patients (*n* = 231) vs placebo (*n* = 100)	VEGFR, KIT, RET, EGFR	30.5 months, HR: 0.46 (0.31–0.69), *P* < 0.001	HR: 0.89 (0.48–1.65)	Diarrhoea (56%), rash (45%), nausea (33%), hypertension (32%) and headache (26%)	2011
Cabozantinib (EXAM study) (NCT00704730)	3	Advanced or metastatic MTC (*n* = 330)	Cabozantinib-treated MTC patients (*n* = 220) vs placebo (*n* = 110)	VEGFR2, KIT, FLT3, AXL, MET, RET	11.2 months, HR: 0.28 (0.19–0.40), *P* < 0.0001	44.3 months, HR: 0.85 (0.64–1.12), *P* = 0.24, 44.3 months, * RET M918T + *HR: 0.60 (0.38–0.94), *P* = 0.03	Fatigue, diarrhoea, decreased appetite, nausea, weight loss and palmar-plantar erythrodysaesthesia, no cardiological AEs	2012
Lenvatinib (NCT01728623)	2	Advanced or metastatic MTC (*n* = 9)	Lenvatinib-treated MTC patients (*n* = 9)	VEGFR, KIT, RET, PDGFR, FGFR	9.2 months (1.8–NR)	12.1 months (3.8–NR)	Hypertension (89%), palmar-plantar erythrodysaesthesia (89%), diarrhoea (89%)	No
Motesanib (NCT00121628)	2	Advanced MTC (*n* = 9)	Motesanib-treated MTC patients (*n* = 91)	VEGFR1,2,3, PDGFR, KIT	4 months, HR:13 (13–13.2)	NA, at 12 months: 75% of patients survived	Diarrhoea (41%), fatigue (41%), hypothyroid (29%), hypertension (27%) and anorexia (27%)	No
Sunitinib (NCT00519896)	2	Iodine-refractory WDTC or MTC (*n* = 7) (total thyroid cancer, *n* = 33)	Sunitinib-treated MTC patients (*n* = 7)	VEGFR1,2, PDGFR, c-KIT, FLT3, RET	12.8 months (for all thyroid cancers)	Not reached	Fatigue (11%), neutropaenia (34%), palmar-plantar erythrodysaesthesia (17%), diarrhoea (17%), leukopenia (31%)	No
Imatinib (with dacarbazine and capecitabine) (NCT00354523)	2	Advanced or metastatic MTC (*n* = 15)	Imatinib-treated MTC patients (*n* = 15)	Bcr-Abl, (PDGFR)α, PDGFRβ, c-Fms, c-Kit, RET	7/15 pts: 3 months (2–8)	NA	Hypothyroid (33%), rash, malaise, and laryngeal mucosal swelling (12%), serious haematological toxicity (grade 3) (6%)	No
Anlotinib (ALTER 01031)(NCT02586350)	2B	Advanced or metastatic MTC not previously treated with antiangiogenetic target therapy (*n* = 62)	Anlotinib-treated MTC patients (*n* = 62) vs placebo (*n* = 29)	VEGFR, FGFR, PDGFR, c-Kit	20.67 (14.03–34.63) vs 11.07 (5.82–14.32), (HR: 0.53, *P* = 0.0289)	ND	Palmar-plantar erythrodysaesthesia, hypertension, hypertriglyceridaemia and diarrhoea	No
Pazopanib (NCT00625846) (completed 2020)	2	Advanced thyroid cancer (MTC, *n* = 35)	Pazopanib-treated MTC patients (*n* = 35)	VEGF1,2, PDGFa,b, FGF, ITK	6 months, 0.686 (0.548–0.858)	NA	Weight loss (11.4%), depression (8.6%), abdominal pain (5.7%), skin rash (5.7%)	No
Sorafenib (NCT00390325) (ongoing)	Systematic review of 8 studies/ongoing trial	Advanced MTC *n* = 101/MTC *n* = 21 (active but no recruitment)	Sorafenib-treated MTC patients (*n* = 101)/not completely	Raf serine/threonine kinases, VEGFR1,2,3 (PDGFRβ)	14.5 (12.4–16.3)/no data yet	ND	Palmar-plantar erythrodysaesthesia (69%), diarrhea (49%), hypertension (35%), skin rash (39%), fatigue (39%)	No
Selpercatinib (LOXO-29) (NCT03157128)	1/2	RET-mutant or not MTC patients (*n* = 143)	*RET*-mutant MTC patients treated (*n* = 55) vs not previously treated (*n* = 88) with vandetanib or cabozantinib	- ATP-competitive small molecule *RET* inhibitor - Inhibit different *RET* alterations, including the V804M mutation - Low activity on VEGFR	1-year-PFS: 82% (69–90) in the* RET*-mutant MTC pre-treated and 92% (82–97) in not previously treated patients	ND	Hypertension (43%), diarrhoea (38%), fatigue (38%)	2020
Pralsetinib (BLU-667) ARROW trial (NCT03037385)	1/2	RET-mutant MTC patients (*n* = 84)	RET-mutant MTC patients treated (*n* = 61) vs not previously treated (*n* = 23) with vandetanib or cabozantinib)	- 10× potency over other MKIs against *RET* variants and resistance mutants - No VEGFR inhibition	- Median PFS: not reached - Estimated 1-year PFS: 75% (63–86) in *RET*-mutant pre-treated and 81% (63–98) in not previously treated patients	- Median OS : not reached - Estimated 1-year OS: 89% (81–97) in *RET*-mutant pretreated and 91% (78–100) in not previously treated patients	Grade 3: hypertension (40%), diarrhoea (38%), fatigue (35%) and anaemia, lymphopaenia, neutropaenia	2020
Axitinib (NCT00094055)	2	Advanced or metastatic thyroid cancer (total *n* = 60; MTC, *n* = 11)	Axitinib-treated MTC patients (*n* = 11)	VEGFR1,2,3	- 18.1 months (for all thyroid cancers)^a^ - MTC patients: 2/11 had PR, 3/11 had SD	Not reached	Grade > 3: hypertension (*n* = 7; 12%)	No

^a^No separated data for MTC.

AE, adverse events; EGFR, epithelial growth factor; FLT3, FMS/KIT-like gene; HR, hazard ratio; KIT, proto-oncogene, receptor tyrosine kinase; MTC, medullary thyroid cancer; NA, not available; ND, no data; NR, not reported; OS, overall survival; PDGFR, podocyte growth factor receptor; PFS, progression-free survival; PR, partial response; RET, rearranged during transfection; SD, stable disease; TKI, tyrosine kinase inhibitors; VEGFR, vascular epithelial growth factor; WDTC, well differentiated thyroid cancer.

The first approved TKI, vandetanib, selectively targets *RET*, vascular epithelial growth factor receptor (VEGFR) and EGF receptors (EGFR) (Wedge *et al.* 2002, Wells *et al.* 2012). The efficacy of vandetanib was evaluated in 331 individuals with progressive MTC (123 had tumour progression, either locoregional or distant metastases) randomised 2:1 to receive vandetanib (300 mg/day) or placebo (Zactima Efficacy in Thyroid Cancer Assessment(ZETA) study) (Wells *et al.* 2012). Inclusion criteria included measurable, unresectable locally advanced or metastatic, hereditary or sporadic MTC. The results showed a statistically significant increase in the PFS in the vandetanib-treated group compared to placebo (30.5 vs 19.3 months, *P*  < 0.001), as well as a higher objective response rate. Vandetanib has also been successfully used to treat children with MEN2B (Fox *et al.* 2013).

The second approved TKI, cabozantinib (140 mg/day), is a c-MET, VEGFR2 and RET inhibitor. A randomised (2:1) study of 330 individuals demonstrated a significant increase in PFS in the cabozantinib-treated group compared to placebo (11.2 vs 4.0 months, *P*  < 0.001) in the advanced MTC (EXAM) study (Elisei *et al.* 2013). In this trial, all patients had documented radiological disease progression according to RECIST (40% had received previous anti-cancer therapies, including other TKIs). The effect of vandetanib or cabozantinib on the overall survival of MTC patients remains unknown, but interim analyses have not revealed any difference between the experimental and placebo arms (Wells *et al.* 2012, Elisei *et al.* 2013). This could potentially be due to the cross-over design in the ZETA study, but patients receiving placebo were not allowed to cross-over in the EXAM study. *In vivo* data have shown that both drugs inhibit angiogenesis in a dose-dependent manner, although cabozantinib showed more potent anti-angiogenic activity than vandetanib (Carra *et al.* 2021). In a recent retrospective study including MTC patients (*n*  = 48) with locally advanced disease and/or evidence of distant metastases treated with vandetanib and/or cabozantinib (as first-line and later-line treatment), the median PFS for first-line vandetanib- and cabozantinib-treated patients was 12 and 9 months, respectively. The median total OS (for the total duration of treatment, first-line and later-line settings) was 54 and 24 months, respectively (Koehler *et al.* 2021); however, these results are difficult to interpret given that significantly more patients received vandetanib in the first-line setting (85%) compared with cabozantinib (15%), and this likely accounts for the poorer prognosis of cabozantinib-treated patients. In vandetanib-treated patients, the PFS and OS were significantly longer in patients aged ≤60 years at TKI initiation and in patients with ≥5 treatment-related adverse events. In addition, the PFS was prolonged in those without bone metastases (Koehler *et al.* 2021).

The genetic signature is crucial for predicting responsiveness to therapy in progressive MTC. Phase 3 trials have helped identify predictors of response to both vandetanib and cabozantinib. The subgroup of patients in the ZETA study with the *RET M918T* mutation showed a higher response rate to vandetanib than *RET M918-*negative patients (54.5% vs 32%), but these data were based on a small sample size (Wells *et al.* 2012). In a larger phase 3 trial, cabozantinib appeared to prolong PFS vs placebo in the *RET* mutation-positive subgroup of patients with radiologically documented progressive MTC (HR: 0.23; 95% CI: 0.14–0.38; *P*  < 0.0001), in the *RET* mutation-unknown subgroup (HR: 0.30; 95% CI: 0.16–0.57; *P* = 0.0001) and in the *RAS* mutation-positive subgroup (HR: 0.15; 95% CI: 0.02–1.10; *P* = 0.0317). The *RET M918T* subgroup achieved the greatest observed PFS from cabozantinib compared to placebo (HR: 0.15; 95% CI: 0.08–0.28; *P*  < 0.0001). Furthermore, the PFS following cabozantinib was prolonged in patients with *RET M918T* (61 vs 17 weeks) or *RAS* mutations compared to patients with other mutations (Sherman *et al.* 2016). A microRNA expression analysis in MTC has shown increased expression of miR-375 and decreased protein levels of the miR-375 target SEC23A in MTC, both of which conferred increased sensitivity to vandetanib. *In vitro* studies have shown that *RET* codon 804 and 806 mutations confer resistance to vandetanib therapy (Carlomagno *et al.* 2009).

Common adverse effects of TKIs include hypertension, palmar–plantar erythrodysaesthesia, proteinuria and QTc interval prolongation, with the most prominent being fatigue, lethargy, anorexia and gastrointestinal disturbances; however, all TKIs have a unique adverse event profile (Table 1). Approximately 35% of the patients treated with vandetanib had a QTc prolongation of more than 60 ms, which is considered a grade 4 adverse event requiring dosage reduction, but this was not encountered in the phase 3 cabozantinib trial (Schlumberger *et al.* 2017). Fistulas in the gastrointestinal tract are rare (<1%), although extremely dangerous, adverse events associated with cabozantinib. Practical guidelines on the management of the more common adverse events have been developed (Rao & Cabanillas 2018, Cabanillas & Takahashi 2019). Patients need to be carefully assessed, focusing on their performance status along with the burden and location of metastases. After treatment commencement, regular review is required to allow for the early and aggressive management of potential adverse events. In cases of less serious adverse events (grade 1–2), dose modification, by lowering the administered dose, should be considered in parallel with symptomatic treatment; however, in cases of serious adverse events (grades 3–4), treatment should be interrupted or suspended.

In the case of progression on an initial TKI, treatment with another TKI, besides those currently approved, may be considered when accessible. Lenvatinib is a TKI inhibiting VEGFR-1, 2, and 3, fibroblast growth factor receptor (FGFR)-1–4, platelet-derived growth factor receptor (PDGFR), *RET* and KIT signalling pathways, that has been approved for the treatment of differentiated thyroid cancer, but only scanty data exist on its use in MTC. In a phase 2 study including 59 patients with unresectable progressive MTC, disease control was achieved in 80% (95% CI: 67–89%), 44% of whom had stable disease (Schlumberger *et al.* 2016). Median PFS was 9.0 months (95% CI: 7.0 – not evaluable). Similarly, in a smaller but more recent phase 2 trial of 51 patients with all histological types of progressive thyroid cancer, including 9 patients with MTC, the median PFS for MTC patients was 9.2 months, with a 22% response rate (Takahashi *et al.* 2019). Lenvatinib can induce posterior reversible encephalopathy syndrome, which in combination with Takotsubo cardiomyopathy resulted in a fatal outcome in one case (Chae *et al.* 2018). Other rarer adverse events include thrombocytopaenia, fistula formation and heart failure (Cabanillas & Takahashi 2019).

Sorafenib is a dual inhibitor, targeting Raf pathways as well as tyrosine kinase pathways including VEGFR-1, VEGFR-2, VEGFR-3 and PDGFR-β. In two meta-analyses including 101 and 99 progressive MTC patients, respectively, from 8 studies using sorafenib as the single TKI treatment (previous treatments included surgery, chemotherapy or radiotherapy), sorafenib showed an intermediate efficacy. In the first meta-analysis, an overall partial response and stable disease were found in 21 and 58% of MTC patients, respectively (Vuong *et al.* 2019), while in the other, an objective response (including complete and partial responses) was found in 27.6% of patients with a PFS of 12.4 months (95% CI: 8.4–16). However, this was associated with a high discontinuation rate of up to 32.3% (95% CI: 24.3–40) of patients due to toxicity (Vuong *et al.* 2019, Efstathiadou *et al.* 2021). Responses to sorafenib were not durable, with resistance developing, usually within 1–2 years (Vuong *et al.* 2019).

Anlotinib is a TKI that inhibits both tumour angiogenesis and tumour cell proliferation by blocking VEGFR, FGFR, PDGFR and c-Kit simultaneously (Shen *et al.* 2018, Li *et al.* 2021). In a single-arm phase 2 study conducted in China, 58 patients with progressive MTC (unresectable, locally advanced or metastatic MTC) treated with anlotinib exhibited an objective response rate (ORR) of 56.9% (Sun *et al.* 2018), with a PFS rate at 48 weeks of 85.5%. The most common adverse events were palmar-plantar erythrodysaesthesia, hypertriglyceridaemia, cholesterol elevation, fatigue and proteinuria.

In a recent meta-analysis including 33 studies (23 prospective, 9 retrospective, 1 observational) with 99 metastatic MTC patients and 16 cohorts showing progressive disease *ab* *initio*, overall stable disease was observed in 46.2% of TKI-treated patients and progressive disease in 22.9% (Efstathiadou *et al.* 2021). In particular, the use of TKIs conferred a PFS of 23.3 months (95% CI: 21.07–25.5). The meta-analysis included patients treated with various TKIs including axitinib, cabozantinib, dovinitib, imatinib, lenvatinib, motesanib, pazopanib, sorafenib, sunitinib, vandetanib and combinations of these treatments. In particular, progressive disease occurred in 23.7% of patients treated with vandetanib, in 22.6% treated with cabozantinib and in 19.3% treated with sorafenib. Grade 3 or greater adverse events occurred in 48.5% of TKI-treated patients and drug discontinuation was reported in 44.7%. Vandetanib induced an objective response in 33.8% and cabozantinib in 27.7% of MTC patients (Efstathiadou *et al.* 2021).

A limitation of TKI treatment is that the tumour cells eventually develop resistance, associated with tumour progression, independent of the type of TKI used or molecular tumour profile. Secondary mutations in the kinase domains that inhibit the binding of TKIs, usually downstream from the TKI target, resulting in a mechanism that bypasses the action of the drug (Viola *et al.* 2016). In MTC patients, acquired *RET V804M* ‘gatekeeper’ resistance mutations have been described in vandetanib-treated patients (Subbiah *et al.* 2018*a*). In such cases, a second TKI such as cabozantinib that acts *via* more than one molecular pathway might be considered in order to counteract such resistance (Priya *et al.* 2017). Of note, the discontinuation of TKI treatment could lead to a rapid increase in tumour growth and disease progression (Resteghini *et al.* 2017), presumably because the TKI blockade was being partially (but only partially) reversed by the activation of alternative signalling pathways. In such a situation, combination drug use may be considered, although this approach is investigational (see below).

#### Specific inhibitors of RET oncogene alterations

In 2020, two new RET-selective inhibitors were FDA-approved: selpercatinib (LOXO-292) and pralsetinib (BLU-667), with the former now also having been approved by the EMA.

Recently, selpercatinib (LOXO-292), which *selectively* targets oncogenic *RET* alterations, was approved by the FDA in the United States for the treatment of *RET-*mutant MTC and *RET* fusion thyroid cancers (Wirth *et al.* 2020); it has recently also been approved in the United Kingdom in patients resistant or intolerant of the multikinase inhibitors. In 55 *RET*-mutant MTC patients who had previously progressed through or not tolerated vandetanib and/or cabozantinib, the objective response rate was 69% (95% CI: 55–81), and the 1-year PFS was 82% (95% CI: 69–90). In 88 *RET*-mutant MTC patients who had not previously received vandetanib or cabozantinib, the response rate was 73% (95% CI: 62–82), and the 1-year PFS 92% (95% CI: 82–97). The most common adverse events of grade 3 or greater were hypertension in 21% of patients, increased alanine aminotransferase (ALT) and aspartate aminotransferase (AST) levels in 11 and 9% respectively, hyponatraemia in 8% and diarrhoea in 6%. Similar response rates were observed in 3 out of 11 patients (27%) who harboured the *RETV804M* mutation, which confers acquired resistance to vandetanib, showing clinical and radiological responses with a maximum change in tumour size ≥60% after receiving selpercatinib, although resistance eventually developed (Wirth *et al.* 2020).

Pralsetinib (BLU-667) is also a highly selective *RET* inhibitor (Subbiah *et al.* 2018*a*). Pralsetinib potently inhibits the growth of thyroid cancer xenografts driven by various *RET* mutations and fusions, without inhibiting VEGFR-2. In a phase 2 study (NCT03037385) including MTC patients with documented radiological progression, 122 patients with *RET*-mutant MTC (61 patients pre-treated with cabozantinib or vandetanib, or both, and 23 treatment-naïve patients) showed an overall objective radiological response rate of 71% (95% CI: 48–89) by RECIST criteria in treatment-naive patients and 60% (95% CI: 46–73) in pre-treated patients (Subbiah *et al.* 2018*b*). The median PFS and OS were not reached, but the estimated one-year PFS after a median follow-up of 15 months was 75% (95% CI: 63–86) in pre-treated patients and 81% (95%CI: 63–98) in treatment-naïve patients. Overall, pralsetinib appeared to be well-tolerated. The most common adverse event was grade 1 constipation (23%). Grade 3 adverse events included hypertension (8%) and neutropaenia (4%), but there were no grade 4/5 adverse events (Subbiah *et al.* 2018*b*).

In 2020, both the new generation TKIs, selpercatinib and pralsetinib, were granted FDA approval in patients with advanced *RET*-mutant MTC who require systemic therapy and indeed they are considered a highly potent treatment in patients with *RET*-driven MTCs (Subbiah *et al.* 2018*a*). However, there is concern that these highly selective inhibitors may not be as ‘durable’ as the multikinase inhibitors, with resistance being seen to occur more rapidly as follow-up studies progress (Lin *et al.* 2020, Lin & Gainor 2021).

The vast majority of MTC cases resistant to selpercatinib or pralsetinib are *RET*-mutant negative; further investigation of *RET*-independent resistance mechanisms has demonstrated that the resistance to selective *RET* inhibition may be driven by acquired *MET* or *KRAS* amplifications (Lin *et al.* 2020). In the cases of resistance in which the RET pathway is involved, RET solvent front (G810) mutations as well as hinge region (Y806) mutations confer selpercatinib resistance through steric hindrance with drug binding (Lin *et al.* 2020, Solomon *et al.* 2020, Subbiah *et al.* 2021). Moreover, using X-ray crystal structures of RET–selpercatinib and RET–pralsetinib complexes, these inhibitors are not susceptible to* RET* gatekeeper (V804) mutations because of their mode of binding, while remaining susceptible to non-gatekeeper mutations (Subbiah *et al.* 2021). In addition, gatekeeper mutations have been more readily identified after progression on approved TKIs in EGFR-mutant or ALK fusion-positive non-small-cell lung cancer (NSCLC) as compared with* RET* fusion-positive NSCLC (Lin *et al.* 2020). It will be necessary to develop *RET* inhibitors with maintained potency against *RET* resistance mutations, and it may be that combination strategies will be needed to effectively overcome resistance in these patients. As an example, TPX-0046 is a potent RET/SRC inhibitor which has demonstrated preclinical potency against *RET* G810 solvent front mutations and is currently in phase 1 testing in patients with advanced *RET-*altered solid tumours (NCT04161391).

#### When to initiate TKIs and what TKI to choose

The initiation of systemic treatment with TKIs is recommended in the setting of progressive or extensive distant metastases. The current recommendation (Wells *et al.* 2015, Haddad *et al.* 2018) is to consider treatment for tumours >1–2 cm in diameter growing >20% per year, or for symptomatic control: clearly depending on availability, one could use the selective *RET*-antagonists for germline or somatic *RET*-mutation-positive tumours and vandetaninib or cabozantinib when these are absent. However, in the United Kingdom, the more selective RET inhibitors are currently only funded when patients progress through the multikinase inhibitors or when these cannot be tolerated, even after dose reduction. It may be that the superior adverse-event profile of pralsetinib and selpercatinib will determine their first-line use, availability and expense allowing, but as yet there is a question mark over their response durability, and longer trial follow-up is awaited. It is also unclear as to whether the presence of a *RET* mutation, either germline or somatic, is mandatory before the use of either the multikinase or the *RET* selective inhibitors. TKIs in MTC patients with progressive disease are associated with a moderate therapeutic benefit, including disease stability or partial responses in up to 73% of cases, decreasing the 10-year disease-specific mortality rate from 38 to 13.5%, mainly seen in patients harbouring *RET* M918T mutations (Dvorakova *et al.* 2008, Taccaliti *et al.* 2011, Kuo *et al.* 2018). While the toxicity of these drugs is not negligible, it is usually manageable.

### Investigational therapy

#### Combined targeted therapies

Certain small molecule inhibitors possess efficacy against MTC *in vivo* in combination with other drugs (Priya *et al.* 2017). Sunitinib and cisplatin cooperatively interfere with the autophagic lysosomal pathway, and this combination appears to be active in metastatic or progressive MTC (Lopergolo *et al.* 2014). Acquired resistance to targeted therapy could possibly be overcome by employing a combination of targets such as everolimus plus 5-aza-2′-deoxycytidine (AZA), as this combination was found to have a synergistic effect even in everolimus-resistant cell lines (Vitale *et al.* 2017). In other neuroendocrine tumours, combination therapies may show synergistic effects, at least *in vitro* (Aristizabal Prada *et al.* 2018).

In a recent meta-analysis (Funakoshi *et al.* 2014), the combination of cytotoxic chemotherapy and various TKIs (vandetanib, cabozantinib, sorafenib and sunitinib) exhibited a prolonged PFS (HR: 0.82, 95% CI: 0.76–0.89) but had no effect on OS (HR: 0.99, 95% CI: 0.95–1.03) compared to chemotherapy alone in a variety of malignancies, including MTC. However, compared with chemotherapy alone, the addition of a TKI significantly increased the risk of any AEs (RR: 1.63, 95% CI: 1.32–2.01), treatment discontinuation (RR: 1.80, 95% CI: 1.58–2.06) and any severe AEs (RR: 1.25, 95% CI: 1.16–1.36).

#### Monotherapy agents and epigenetic therapy

Nelfinavir (NFV), a heat shock protein (HSP)-90 chaperone, is required for the folding and stability of *RET* mutants (Alfano *et al.* 2010). HSP-90 overexpression is found in a significant proportion of MTCs and may correlate with metastatic potential and *RET* mutations. HSP90 is a molecular target for the HIV-protease inhibitor NFV. It has been shown to have a wide spectrum of activity *in vivo* against MTC cells and may emerge as an important therapeutic option (Valea & Georgescu 2016).

#### mTOR inhibitors

Given that *RET* and RAS activate the PI3K/AKT/mTOR pathway, the anti-proliferative activity of everolimus, an inhibitor of mechanistic target of rapamycin (mTOR) approved for the treatment of neuroendocrine tumours and renal cell carcinoma, was demonstrated in an MTC human cell line (Grozinsky-Glasberg *et al.* 2010) and some activity in a single patient with an MTC (Druce *et al.* 2012). In a small phase 2 trial, seven patients with progressive metastatic or inoperable MTC (Schneider *et al.* 2015) were treated with everolimus. The median PFS was 33 weeks, but no objective response was observed (Schneider *et al.* 2015). Similar findings were encountered in another phase 2 trial that included nine MTC patients treated with everolimus (Lim *et al.* 2013). The median PFS was not reached (after a median follow-up of 11 months), indicating that everolimus may have activity against MTC and that larger prospective studies are warranted. Biochemical response, defined as calcitonin and CEA levels ≥50% lower than baseline, was observed in three (30%) and four (44%) patients, respectively. The most common treatment-related AEs were mucositis, anorexia and AST/ALT elevation, and were in general mild to moderate (Lim *et al.* 2013).

Nevertheless, promising experimental data have been reported in MTC-1.1 cells exposed to combination treatment including everolimus and sorafenib or sunitinib (Lin *et al.* 2012). Everolimus increased the anti-proliferative effects of sunitinib and sorafenib by 24 and 27%, respectively, in MTC-1.1 cells and by 20 and 23%, respectively, in thyroid papillary (TT) cells (Lin *et al.* 2012).

In another report, everolimus was prescribed in addition to vandetanib in a patient who developed disease progression, resulting in a 25% reduction of tumour volume for 8 months (Heilmann *et al.* 2016). The combination of *RET* kinase inhibitors and mTOR inhibitors might be an interesting dual-targeting strategy, but further evaluation in larger clinical trials is mandatory for establishing the most appropriate therapeutic regimen. Most importantly, such combinations, while theoretically attractive and soundly-based, may lead to a compounding of adverse effects rendering them clinically impractical.

#### Immunotherapy

Over the past few years, immunotherapy has been established as an oncological treatment for several types of malignancies, and preclinical studies on MTC have revealed potential benefit (Naoum *et al.* 2018). Data on clinical studies in MTC are scarce, but in a single phase 1 study including a patient with MTC, blocking the PD-1/PD-L1 interaction using nivolumab, an anti-PD-1 agent, resulted in a partial response (Yamamoto *et al.* 2017). Another case report also described a patient with MTC already treated with sunitinib and, having undergone a 3-month trial with the GI-6207 cancer vaccine, was enrolled in a phase 1 trial with the PD-L1 inhibitor avelumab (NCT01772004). Following treatment, he developed stable disease along with a >40% decrease in his calcitonin level (Del Rivero *et al.* 2020). Very preliminary results of a phase 2 trial (NCT03246958) evaluating nivolumab plus ipilimumab in seven patients with progressive MTC and prior TKI failure showed a lack of objective response in all seven patients, although without providing any further specific detail (Lorch *et al.* 2020).

It is well known that high tissue tumour mutational burden (TMB) is a useful marker to recognise patients with recurrent or metastatic advanced solid tumours who may show a better therapeutic benefit from immune checkpoint inhibitors. Therefore, the NCCN guidelines for MTC management suggest that pembrolizumab should be considered in patients with symptomatic, progressive MTC with high TMB ≥ 10 mutations/megabase (NCCN 2021). MTC is reported to show low PD-L1 expression in both tumour cells and tumour-infiltrating immune cells and no microsatellite instability (Bongiovanni *et al.* 2017, Bi *et al.* 2019), irrespective of the presence or absence of either desmoplasia, lymph node metastases and/or *RET* mutation (Bai *et al.* 2019, 2020, Shi *et al.* 2019, Ingenwerth *et al.* 2020). Nevertheless, a cohort of 201 consecutive Chinese patients with MTC (Shi *et al.* 2019) demonstrated that PD-L1 positivity was associated with more aggressive clinico-pathologic features, such as larger tumour size, number of lymph node metastases and advanced TNM staging and was independently predictive of morphological and/or biochemical recurrence or persistent disease (Sørlie *et al.* 2003). Similarly, PD-L1 positivity found in approximately 20% of surgical specimens (although very faint or moderate staining) significantly correlated with distant metastases at surgery (Bi *et al.* 2019). The correlation between PD-1/PD-L1 status and prognosis suggests that immunotherapy targeting PD-L1/PD-1 might indeed be effective in treating advanced MTC under some circumstances.

Five registered clinical trials are currently active studying immune-checkpoint inhibitors in progressive MTC patients (Table 2). Pembrolizumab (NCT03072160), nivolumab and ipilimumab (NCT03246958) are currently being evaluated in phase 2 trials for the treatment of recurrent or metastatic MTC. Interestingly, among the registered studies, there are also trials evaluating the combination of TKI agents together with immunotherapy or chemotherapy as a novel therapeutic option in the treatment of advanced MTC, although the combination of immunotherapy and chemotherapy is well established in other areas of oncology (Table 2).

**Table 2:** Registered (clinicaltrials.gov) clinical trials for the treatment of advanced and /or metastatic MTC.

Clinical trial	ID	Molecule tested	Status of the study	Type of the study (phase)
**MKIs**				
**Phase 1 and 2**				
- A Study of LOXO-292 in Participants With Advanced Solid Tumours, RET Fusion-Positive Solid Tumours and MTC	NCT03157128	Selpercatinib (LOXO-292)	Recruiting	1 and 2
- Study of TPX-0046, a RET/SRC Inhibitor in Adult Subjects With Advanced Solid Tumours Harbouring RET Fusions or Mutations	NCT03647657	TPX-0046 (third generation, highly selective TKI)	Recruiting	1 and 2
- A Study of HA121-28 Tablets in Patients With MTC	NCT04787328	HA121-28	Recruiting	2
- A Study Using Regorafenib as Second or Third-Line Therapy in Metastatic MTC	NCT02657551	Regorafenib	Recruiting	2
- A Study Using Regorafenib as Second or Third-Line Therapy in Metastatic MTC	NCT02657551	Regorafenib	Recruiting	2
- Selpercatinib Before Surgery for the Treatment of RET-Altered Thyroid Cancer	NCT04759911	Selpercatinib (LOXO-292)	Recruiting	2
- Sorafenib Tosylate in Treating Patients With Metastatic, Locally Advanced or Recurrent MTC	NCT00390325	Sorafenib tosylate	Active recruitment completed	2
- Sunitinib Malate in Treating Patients With Thyroid Cancer That Did Not Respond to Iodine I^ 131^ and Cannot Be Removed by Surgery	NCT00381641	Sunitinib malate	Active not recruited yet	2
- A Study of Selpercatinib (LY3527723) in Participants With Advanced Solid Tumours Including RET Fusion-positive Solid Tumours, MTC and Other Tumours With RET Activation	NCT04280081	Selpercatinib	Active not recruited yet	2
- Cabozantinib-S-Malate in Treating Younger Patients With Recurrent, Refractory, or Newly Diagnosed Sarcomas, Wilms Tumour, or Other Rare Tumours	NCT02867592	Cabozantinib-S-malate	Active not recruited yet	2
- Pazopanib Hydrochloride in Treating Patients With Advanced Thyroid Cancer	NCT00625846	Pazopanib hydrochloride	Completed (2020) results pending	2
- Safety, Efficacy and Tolerability of BOS172738 in Patients With Advanced Rearranged During Transfection (RET) Gene-Altered Tumours	NCT03780517	BOS172738 (selective RET inhibitor)		2
- Cabozantinib-S-Malate in Treating Younger Patients With Recurrent, Refractory, or Newly Diagnosed Sarcomas, Wilms Tumour, or Other Rare Tumours	NCT02867592	Cabozantinib-S-malate	Active not recruited yet	2
**Phase 3 and 4**				
- A Study of Selpercatinib (LY3527723) in Participants With RET-Mutant MTC	NCT04211337	Selpercatinib, cabozantinib, vandetanib	Recruiting	3
- A Study of Two Different Doses of Cabozantinib (XL184) in Progressive, Metastatic MTC	NCT01896479	Cabozantinib (XL184) 140 mg vs cabozantinib (XL184) 60 mg vs placebo	Active not recruited yet	4
- A Study of Pralsetinib Versus Standard of Care (SOC) for Treatment of RET-Mutated MTC	NCT04760288	Pralsetinib, cabozantinib, vandetanib	Active, not yet recruiting	3
- To Compare The Effects Of Two Doses Of Vandetanib In Patients With Advanced MTC	NCT01496313	Vandetanib 150 vs 300 mg	Active not recruited yet	4
- An Efficacy Study Comparing ZD6474 to Placebo in MTC	NCT00410761	ZD6474 (vandetanib)	Active not recruited yet	3
- A Study of Two Different Doses of Cabozantinib (XL184) in Progressive, Metastatic MTC	NCT01896479	Cabozantinib 60 vs 140mg	Active not recruited yet	4
**ICIs (all phase 1 and 2)**				
- A Phase II Study of Durvalumab (MEDI4736) Plus Tremelimumab for the Treatment of Patients With Progressive, Refractory Advanced Thyroid Carcinoma – The DUTHY Trial	NCT03753919	Durvalumab	Recruiting	2
- A Phase 2 Study of Nivolumab Plus	NCT03246958	Nivolumab+ipilimumab	Active not recruiting	2
- Ipilimumab in RAI Refractory, Aggressive Thyroid Cancer With Exploratory Cohorts in Medullary and Anaplastic Thyroid Cancer				
- Pilot Trial of Nivolumab Plus Cabozantinib for Advanced Solid Tumours in Patients With HIV Infection	NCT04514484	Nivolumab	Recruiting	1
- Phase II Trial of Pembrolizumab in Recurrent or Metastatic MTC	NCT03072160	Pembrolizumab	Completed (results submitted)	2
- Secured Access to Pembrolizumab for Patients With Selected Rare Cancer Types	NCT03012620	Pembrolizumab	Recruiting	2
**PRRT (all phase 1 and 2)**				
- Indium In 111 pentetreotid in treating patients with refractory cancer	NCT00002947	^111^In pentetreotid	Terminated	1
- ^177^Lu-PP-F11N in Combination With Sacubitril for Receptor-Targeted Therapy and Imaging of Metastatic Thyroid Cancer	NCT03647657	^ 177^Lu-PP-F11N	Recruiting	1
- ^177^Lu-PP-F11N for Receptor-Targeted Therapy and Imaging of Metastatic Thyroid Cancer (Lumed)	NCT02088645	^177^Lu-PP-F11N	Recruiting	1
- Radioactive Drug (^177^Lu-DOTATE) for the Treatment of Locally Advanced metastatic or Unresectable Rare Endocrine Cancers	NCT04106843	^ 177^Lu Dotatate	Recruiting	2
**Others**				
- GFRα4 CAR T Cells in MTC Patients	NCT04877613	Single dose of CART-GFRa4 cells vs fludarabine vs cyclophosphamide	Recruiting	1
- Thermal Ablation of Cervical Metastases From Thyroid Carcinoma	NCT04522570	Laser ablation, cryoablation, radiofrequency ablation	Recruiting	n/a
- QUILT-3.006 for Recurrent MTC (vaccine)	NCT01856920	GI-6207 (recombinant *Saccharomyces cerevisiae-CEA* (610D))	Active not recruited yet	2

Inhibitors, ^177^Lu-PP-F11N, ^177^Lutetium-labelled minigastrin analogue; ICI, immune-check point inhibitors; ^111^In, ^111^Indium; MKI, multikinase inhibitors; MTC, medullary thyroid cancer; PRRT, peptide receptor radionuclide therapy.

#### Peptide receptor radionuclide therapy (PRRT)

Several studies targeting somatostatin receptors (SSTRs) with radionuclides have been reported in patients with progressive, inoperable or advanced metastatic MTC (Grossrubatscher *et al.* 2020), using radiopharmaceuticals such as ^90^Yttrium (^90^Y), ^177^Lutetium (^177^Lu) or ^111^Indium (^111^In) (Salavati *et al.* 2016, Parghane *et al.* 2020, Satapathy *et al.* 2020). In a recent review including 11 studies and 117 MTC patients treated with ^90^Y, ^177^Lu or ^111^In, radiological response data showed that 37.6% of patients developed progressive disease, 54.7% stable disease, 5.1% partial response and 2.6% complete response; however, response criteria across the studies were heterogeneous (Grossrubatscher *et al.* 2020). Besides radiological evaluation, biochemical responses based on calcitonin plasma concentrations showed that 37% of the patients had a more than 50% calcitonin decrease (Grossrubatscher *et al.* 2020). In a prospective phase II study (*n*  = 31 patients ), a prolongation of calcitonin doubling-time levels in 58% of patients post-^90^Y-DOTATOC was reported (Iten *et al.* 2007). In general, patients with partial responses, disease stability and a biochemical response showed prolonged survival (Iten *et al.* 2007, Salavati *et al.* 2016, Parghane *et al.* 2020). After 17 years of experience following treatment with ^177^Lu-DOTATATE (Beukhof *et al.* 2019), albeit in a very small group of patients, it was concluded that this treatment could be considered in patients with high uptake on an ^111^In-DTPA-octreotide scan and positive somatostatin receptor-2a (SSTR2a) expression in tumour samples (Beukhof *et al.* 2019). Twenty-one patients with advanced MTC treated with PRRT (^90^Y-DOTATATE, ^90^Y-DOTATOC, ^177^Lu-DOTATATE or ^177^Lu-DOTATOC) achieved a median time-to-treatment-failure interval of 14 months (95% CI: 8–25) and a median OS of 63 months (95% CI: 21, not reached). Predictors of poorer OS included a short calcitonin doubling-time (≤24 months), strong ^18^F-FDG avidity and age ≥60 years (Hayes *et al.* 2021). A larger retrospective series of 43 patients all treated with ^177^Lu-DOTATATE showed a RECIST partial response in 4%, stable disease in 58% and a median PFS of 24 months (Parghane *et al.* 2020).

PRRT is often well tolerated but is not free of adverse effects such as nausea, asthenia and elevation of liver enzyme levels, although they are usually mild. Serious complications, such as radiation nephrotoxicity and treatment-related myeloid neoplasms, are relatively rare especially with the more frequently used ^177^Lu-labelled radiopeptides (Brabander *et al.* 2017, Parghane *et al.* 2020, Sonbol *et al.* 2020). Adverse events of all grades of nephrotoxicity were reported in 0–1.3% of patients, with grade 3–4 in 0.8%, and grade 3–4 haematological toxicity in up to 6.5% of cases (Grossrubatscher *et al.* 2020, Parghane *et al.* 2020). Proximal tubular reabsorption of the radiopeptide leads to glomerular fibrosis, which is particularly observed after treatment with ^90^Y-DOTATOC (Parghane *et al.* 2020), and thus the ^177^Lu isotope is usually preferred over ^90^Y. Cumulative and per-cycle renal uptake dose, age, presence of hypertension, diabetes mellitus and previous chemotherapy with nephrotoxic agents could potentially accelerate the decrease in renal function after PRRT. Considering these risk factors, one can modify the treatment plan or change the choice of radiopeptide based on the overall tumour burden (Rottenburger *et al.* 2020).

The use of a concomitant radiosensitising agent, such as low-dose oral capecitabine, with ^177^Lu-DOTATATE, might be also beneficial, as suggested by recent studies reporting up to an 86% disease control rate (Grossrubatscher *et al.* 2020, Satapathy *et al.* 2020). However, the only prospective, randomised study (CONTROL NETs), though non-comparative, of PRRT with radiosensitising chemotherapy (capecitabine/temozolomide) in metastatic midgut NETs showed a similar PFS rate at 15 months compared to PRRT alone (Pavlakis *et al.* 2020). The objective response rate was numerically higher in the combination arm but at the expense of greater G3/4 toxicity.

There is recently *in vitro* and *in vivo* evidence that somatostatin receptor subtype-2 (SSTR2) antagonists are better tools to target neuroendocrine tumours than SSTR agonists (Reubi *et al.* 2017). Using SSTR2 *in vitro* autoradiography on MTC tissue sections, Reubi *et al.* found that ^125^I-JR11 was associated with a significantly higher labelling intensity compared to the agonist ^125^I-Tyr^3^-octreotide. A small pilot study including four patients with advanced neuroendocrine tumours found that ^177^Lu-DOTA-JR11 delivers tumour doses that are approximately 3.5 times higher than those obtained with ^177^Lu-DOTATATE (Wild *et al.* 2014).

While meta-iodobenzylguanidine (MIBG) labelled with ^123^Iodine is most effectively used in diagnostic scintigraphy, radio-labelled ^131^I attached to MIBG is mainly used as a therapeutic, with uptake found in up to 35% MTC (Pasieka *et al.* 2004). Concerning the efficacy of ^131^I-MIBG treatment in MTC, data are limited to case reports and evidence from a prospective randomised trial is lacking (Mukherjee *et al.* 2001, Sze *et al.* 2013). This radionuclide may in general be more myelotoxic (Sze *et al.* 2013). CEA targeting using murine or chimeric anti-CEA bispecific antibodies and pre-targeted hapten-peptides labelled with ^111^In or ^131^I has been also developed for radioimmunotherapy in relapsed MTC (Bodet-Milin *et al.* 2016) (Table 2).

The cholecystokinin-2 receptor (CCK2R or gastrin receptor) is over-expressed in several tumour types, including MTC. CCK2R targeting shows promise for MTC imaging and represents, together with SSTR ligands, potential theranostic approaches in selected cases (Refardt *et al.* 2021). Many peptide-based radiopharmaceuticals for targeting CCK2R have been developed (Behr & Béhé 2002). In a recent study, the ^177^Lu-labelled minigastrin analogue ^177^Lu-PP-F11N showed promising results in six patients with advanced MTC (Rottenburger *et al.* 2020). Recently, a new highly metabolically stable minigastrin analogue (DOTA-MGS5) has shown excellent targeting properties in preclinical studies, supporting its possible translation into clinical trials (Klingler *et al.* 2019).

### Other systemic or local therapies

#### Chemotherapy

The objective response rate of MTC patients to classical cytotoxic chemotherapy such as dacarbazine and 5-fluorouracil is 10–20%, with most studies performed on limited numbers of patients and without robust evaluation criteria such as RECIST (Hadoux & Schlumberger 2017). Long-term responses are uncommon.

#### External beam radiotherapy

External beam radiotherapy (EBRT) may be recommended to improve loco-regional control in patients at high risk of relapse in the neck. The usual indication for EBRT is macroscopically unresectable tumour in a patient older than 45 years of age. EBRT is indicated to improve the local control of disease in cases of local recurrence or loco-regional lymph node metastases, or as palliative therapy to reduce pain from bone metastases or to treat brain metastases (Wells *et al.* 2015). A few studies have shown an improvement in locoregional control but no survival benefit, confirming that although neck disease can be controlled in high-risk patients, the progression of distant disease is still a significant problem (Schwartz *et al.* 2008, Martinez *et al.* 2010, Brierley & Sherman 2012, Call *et al.* 2013, Tsoukalas *et al.* 2017). Localised EBRT and/or bisphosphonate administration should be considered for painful bone metastases or for the prevention of skeletal-related events (Farooki *et al.* 2012). Recommendations from a group with extensive clinical experience suggest quarterly zoledronic acid (Haugen *et al.* 2016). According to ATA guidelines for MTC, adjuvant EBRT can be considered in patients with a high risk of recurrence or with incompletely resected MTC (Haugen *et al.* 2016). However, EBRT was not associated with improved overall or disease-specific survival in a recent study of patients with MTC (Jin *et al.* 2021).

#### Embolisation or cryoablation

Embolisation or cryoablation as a palliative therapy may be beneficial in select cases to decrease tumour burden, pain or refractory diarrhoea associated with liver metastases (Fromigué *et al.* 2006). Radiofrequency thermo-ablation is frequently applicable to bone, liver and lung to treat single metastases or a single progressive and symptomatic lesion in the context of stable disease (Eisele 2016). Another local treatment is the conventional transarterial chemoembolisation (TACE) or radioembolisation (TARE), sometimes used in advanced cases of liver metastatic disease, especially when liver metastases are smaller than 3 cm and liver involvement is less than 30% (Roche *et al.* 2003).

## Hormonal syndromes secondary to MTC

MTC has also been associated with the *ectopic* production of bioactive compounds, peptides or amines, leading to distinct clinical syndromes (Kaltsas *et al.* 2010). The most commonly described ectopic syndrome is ACTH leading to Cushing’s syndrome that in some cases can be severe. Other compounds include serotonin, somatostatin, pro-opiomelanocortin, vasoactive intestinal peptide, chromogranin A, amyloid, gastrin-releasing peptide and histamine (Wells *et al.* 2015)*.* Patients with advanced MTC may experience debilitating diarrhoea and flushing due to hypersecretion of calcitonin, VIP, or increases in intestinal motility (Cox *et al.* 1979, Rambaud *et al.* 1988). Antimotility agents (loperamide or codeine) may be initially used for symptom relief. Further options for refractory cases include somatostatin analogues, although tachyphylaxis typically occurs over time (Zatelli *et al.* 2006). For patients with extensive liver metastases, surgical resection, percutaneous radiofrequency ablation or arterial (chemo)embolisation may be considered in an attempt to reduce the calcitonin levels (Fromigué *et al.* 2006).

Cushing’s syndrome due to ectopic ACTH secretion is found in 0.7% of MTC patients, accounting for 2.2–7.5% of all ectopic ACTH-Cushing’s syndrome cases (Barbosa *et al.* 2005). Fifty cases of MTC-related Cushing’s syndrome have been reported so far. In those cases, the tumour cells are positive for calcitonin but mostly display negative immunostaining for ACTH, presumably due to the high secretion rate (Shahani *et al.* 2010). Ectopic Cushing’s syndrome from MTC is associated with significant morbidity and mortality, as secondary complications of hypercortisolism account for 50% of the mortality in these patients (Hayes & Grossman 2018). In the past, management of hypercortisolism was limited to decreasing metastatic tumour burden and using anti-adrenal therapies such as ketoconazole, mitotane and/or metyrapone. Surgical intervention with bilateral adrenalectomy is another option available to some patients (Hayes & Grossman 2018). However, systemic therapy with tyrosine kinase inhibitors such as vandetanib offers a further management strategy for disease control of metastatic disease and might now be considered a first-line therapy for ectopic Cushing’s syndrome, following control of the excess cortisol, in the setting of unresectable disease or progressive, metastatic disease, as well as for controlling secretory Cushing’s syndrome (Wells *et al.* 2012, Paepegaey *et al.* 2017).

## Future perspectives

In a subset of MTC, octreotide and pasireotide inhibit cell proliferation and invasiveness, especially when the expression of SSRT2 and SSRT5 is high, supporting their potential use in the control of tumour growth. On the contrary, MTC with high SSRT3 expression was unresponsive to both octreotide and pasireotide (Zatelli *et al.* 2006). Calcitonin secretion was not affected by octreotide or by pasireotide in primary cultured MTC cells, regardless of their responsiveness to the SSA’s anti-proliferative effects. These data confirm a dissociation between the anti-mitotic and anti-secretory effects of SSAs in primary cultured MTC cells (Zatelli *et al.* 2006).

Activating transcription factor 4 (ATF4) is a negative regulator of the RET tyrosine kinase receptor in MTC. Low ATF4 protein or ATF4 loss alone had a significant negative impact on median survival compared to high protein expression or diploid ATF4. The combination of somatic *RET* expression and low ATF4 protein levels further decreased overall survival. These data suggest that ATF4 may predict response to tyrosine kinase inhibitors, serve as a prognostic marker for personalised care and represent a therapeutic target in MTC (Williams *et al.* 2021). The small-molecule ONC201 caused apoptotic MTC cell death, decreased transcription of *RET, VEGFR2, IGFBP2* and increased mRNA levels of *ATF4* representing a promising strategy for the treatment of all patients with MTC, including those with TKI-refractory disease and other cancers with *RET* abnormalities (Bagheri-Yarmand *et al.* 2021).

Many novel therapeutic strategies are being pursued to develop personalised treatment approaches in MTC. Inhibitors of alternative targets and pathways, such as aurora kinase inhibitors, mTOR inhibitors, farnesyltransferase inhibitors and exploiting microRNA levels, seem promising (Gild *et al.* 2013, Joo *et al.* 2019). The potential for administering these drugs in a neoadjuvant setting to facilitate surgery and aiming for surgical cure are also on the horizon (Cabanillas *et al.* 2018, Wang *et al.* 2019). Recently, new targets have been highlighted, such as the glucose-dependent insulinotropic polypeptide receptor (GIPR) (Gourni *et al.* 2014) and minigastrin, a ligand targeting cholecystokinin-2 (CCK2) receptors, as noted previously. Ongoing studies (under recruitment or active) are presented in Table 2.

## Conclusions

The introduction of the multikinase inhibitors, together with the recent more selective *RET* inhibitors, has opened a new phase in our treatment of these rare but fascinating tumours. While current response durations are relatively limited, they do suggest the prospect of a gradual improvement in therapies over time, with increasing durability as our understanding of the molecular biology develops. Nevertheless, their efficacy should be further investigated by larger trials with robust methods. In time, we would anticipate a more personalised therapeutic stratagem based on individualised patient tumour profiling and selective signalling pathway targeting. Treatments reducing tumour bulk, such as additional surgery and (chemo)embolisation, as well as external beam radiotherapy, are also utilised, but, generally, the results are not especially encouraging and are best considered as palliative therapies to control symptoms.

## Declaration of interest

The authors declare that there is no conflict of interest that could be perceived as prejudicing the impartiality of this review.

## Funding

This work did not receive any specific grant from any funding agency in the public, commercial or not-for-profit sector.
